# Measurement of Disease Comorbidity Using Semantic Profiling of Disease Genes

**DOI:** 10.3390/ijms26083906

**Published:** 2025-04-21

**Authors:** Seong Beom Cho

**Affiliations:** Department of Biomedical Informatics, College of Medicine, Gachon University, 38-13, Dokgeom-ro 3 Street Namdon-gu, Incheon 21565, Republic of Korea; sbcho1749@gachon.ac.kr

**Keywords:** disease gene, comorbidity, gene-set enrichment analysis, semantic profiling

## Abstract

The identification of overlapping disease genes between different diseases is the first step in the elucidation of the biological mechanism of disease comorbidity; however, in the absence of common genes, it is difficult to determine the mechanism of comorbidity even if clinical evidence of disease co-occurrence exists. In this research, a gene-set-based measurement of the comorbidity of diseases (GS.CoMoD) was proposed. The underlying assumption of GS.CoMoD is that if the *p*-value vectors obtained from the enrichment analyses of different disease gene lists indicate similarity, the diseases are possibly comorbid. Therefore, comorbidity can be detected even without overlapping genes. A simulation analysis showed that GS.CoMoD yielded higher scores for comorbid disease pairs vs. random disease pairs. Moreover, comparison analyses revealed that GS.CoMoD outperformed the pre-existing methods for detecting comorbidity.

## 1. Introduction

Disease comorbidity consists in the coupled occurrence of different diseases [1]. The comorbidity of diseases is determined by testing the co-incidence of two diseases. The relative risk (RR) has been frequently used for the determination of the comorbidity of two diseases [2]. A significant RR indicates that there might be a common molecular mechanism underlying the coupled occurrence of the diseases. The identification of disease comorbidity is achieved using statistical testing of the disease co-incidence rates; however, this only indicates the non-random association of two diseases. Therefore, because it is difficult to understand the molecular mechanism of comorbidity based on these results, information on disease genes that are known to be associated with the development of both diseases simultaneously is required [3].

Understanding the genetic background of diseases is a crucial component of translational research, as it provides information about disease pathophysiology and the identification of drug targets or the building of therapeutic strategies. This is the case with disease comorbidity, and the identification of genes that are associated with this phenomenon is required for understanding the underlying mechanism. For this purpose, the measurement of comorbidity based on overlapping genes has been widely used [4,5,6]. For example, the Jaccard index is a fundamental measure of disease comorbidity that uses information on overlapping genes between two different diseases. The Jaccard index increases as the number of overlapping genes increases; therefore, disease comorbidity can be assumed intuitively. If there are no overlapping genes, the overlapping-gene-based measure reports results of a lack of comorbidity.

The Jaccard index and other types of information, such as gene expression levels, protein–protein interactions, and gene ontology, have been integrated with the overlapping-gene-based measure to provide a biological explanation for comorbidity [5,6,7,8]. For example, Rockson et al. used the Jaccard index to identify comorbid diseases with lymphatic disorders. Menche et al. proposed a network-based overlap of a disease pair, which was determined based on the size of the subgraph on the protein–protein interaction (PPI) network and the mean shortest distance between the genes involved in the two diseases. In turn, Rubio-Perez et al. developed a composite score of unifying common genes and common ontologies between two disease gene sets. These methods exhibit the common characteristics of yielding a higher score as the number of overlapping genes increases. However, it is possible to determine disease comorbidity in the absence of common genes shared by two disease gene sets in the following conditions: First, even if two diseases show comorbidity based on clinical data, common genes might not be available because the disease genes have not been fully discovered. Second, disease comorbidity might occur when different genes of two diseases share common biological functions. To identify the comorbidity of a disease pair, regardless of whether the two diseases have common genes, a unifying frame is necessary.

**Figure 1:**
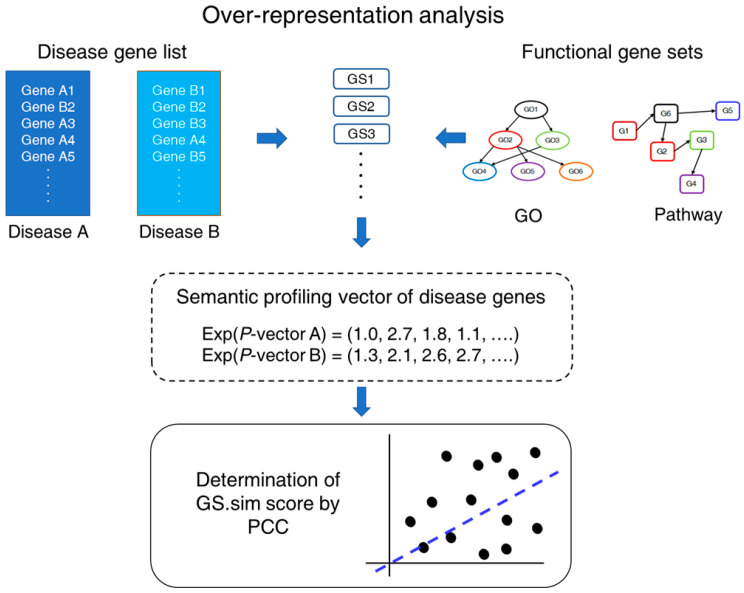
Schematic diagram of GS.CoMoD. For a pair of disease gene lists, gene-set enrichment analyses using Fisher’s exact test (which are termed over-representation analysis here) were performed. From the analysis, two *p*-value vectors were obtained, and exponentials of the vectors were used for the determination of the gene-set-based similarity (GS.sim) score, which is a correlation coefficient between the *p*-value vectors. The functional gene sets indicate gene sets containing genes that are involved in specific functions. GS, gene set; GO, gene ontology; Exp, exponential function; PCC, Pearson’s correlation coefficient.

In this research, a method for the gene-set-based measurement of the comorbidity of diseases (GS.CoMoD) was developed to estimate disease comorbidity using the semantic profiling of disease genes in terms of biological functions (Figure 1). In contrast with previous methods, which used overlapping genes that had been identified as disease genes in different conditions, the present method required no such information; rather, disease genes were tested based on the determination of similarity between the semantic profiling vectors of disease genes. The semantic profiling vector was generated from the test of over-representation of gene sets, which is used for testing the enrichment of specific sets of genes with functional implications [9]. This article is organized as follows: First, detailed explanations about the assumption and analytical processes of GS.CoMoD are provided. Simulation data analysis is then described, aiming to demonstrate the validity of the GS.CoMoD method. Moreover, the results of a comparative analysis of GS.CoMoD with other related methods using disease gene sets and clinical disease co-occurrence data are presented. Finally, the implications and limitations of this study are discussed.

## 2. Results

### 2.1. Identification of Disease Comorbidity Using a Gene-Set Enrichment Analysis

For the determination of the comorbidity of a disease pair using gene information, previous methods have used overlapping genes or proteins between diseases. In contrast, in the present research, GS.CoMoD is proposed for determining the comorbidity of a disease pair based on the semantic profiling vectors of disease genes (Figure 1). Here, the semantic profiling vector is a vector of *p* values that were obtained from a gene-set enrichment analysis of a disease gene list. Because the degree of the *p* values indicates biological functions or themes embedded in the disease gene list, the change in *p* values throughout various functional gene sets represents the functional implications of the disease gene list, which is termed the semantic profiling vector in this research. Therefore, if the *p*-value vectors exhibited similarity, the underlying pathophysiologic mechanism was classified as being similar between the two disease gene lists, which indicates the comorbidity of the diseases.

The first step of GS.CoMoD was the performance of over-representation analyses (ORAs) using different gene sets, such as gene ontology (GO) and pathways. It is well known that the ORA is one of the gene-set methods that affords statistical testing of the over-representation of a gene set in a gene list [9], with a significant result indicating that the genes in a functional gene set appear in the gene list more frequently than expected by chance. Therefore, here, the *p* values of the ORAs indicate whether the gene set is significantly associated with the gene list. Fisher’s exact test was used for the ORAs (Supplementary Methods), yielding two different *p*-value vectors. Subsequently, we performed a computation of the Pearson’s correlation coefficient (PCC) of the *p*-value vectors (Equation (1)). The PCC indicates the degree of similarity among the semantic profiling vectors of two groups of disease genes. Higher positive values indicate greater comorbidity, whereas lower positive values or negative values can be interpreted as the absence of comorbidity of the two diseases. The PCC value was termed the gene-set-based similarity score (GS.sim).

(1) $$GS.sim=\frac{\sum_{i=1}^{n}\left(P_{A}-{\bar{P}}_{A})(P_{B}-{\bar{P}}_{B}\right)}{\sqrt{\sum_{i=1}^{n}{\left(P_{A}-{\bar{P}}_{A}\right)}^{2}}\sqrt{\sum_{i=1}^{n}{\left(P_{B}-{\bar{P}}_{B}\right)}^{2}}}$$

where *A* and *B* are the gene sets of a disease pair, *P_A_* and *P_B_* indicate vectors containing the exponential of *p* values of the gene-set enrichment analysis, and *n* is the number of functional gene sets used in the enrichment analysis. The analytical frame of GS.CoMoD is advantageous in the absence of overlapping genes between two diseases. In this scenario, the overlapping-gene-based measures would reject the presence of the comorbidity of two diseases, as the measure would be zero because of the absence of overlapping genes (Materials and Methods). In contrast, the GS.CoMoD approach can detect comorbidity if two disease gene sets have similar *p*-value vectors, even in the absence of common genes.

### 2.2. Results of Simulation Study

**Figure 2:**
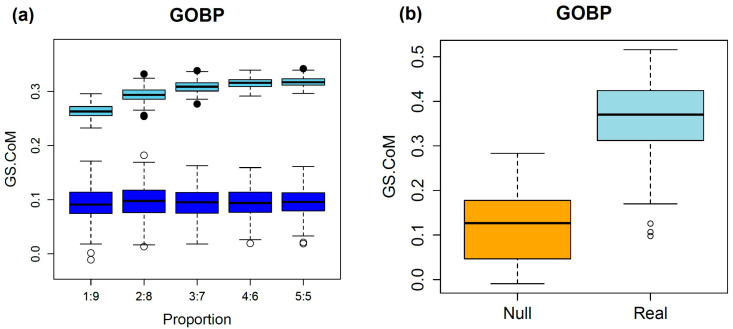
Results of the simulation analysis. (**a**) The upper boxplots are from simulations performed by segregating the disease genes of type 2 diabetes. The *x*-axis indicates the ratios of splitting, and the *y*-axis represents the GS.sim score. The numbers on the *x*-axis are the splitting ratios. For example, 3:7 indicates total disease genes of type 2 diabetes divided into two groups with a ratio of 3:7. The lower boxplots are the results of simulations with the same number of randomly sampled human genes as that of type 2 diabetes genes. The partitioning of the genes was performed in the same manner. (**b**) Comparison of GS.sim score from 100 DisGeNET disease gene sets and random gene sets. Real values indicate scores from the randomly sampled disease gene sets of the DisGeNET database, which have more than 10 genes. Null values include the scores of the gene sets that were randomly selected from the same number of human genes as that of the 100 disease gene sets. All gene sets were divided equally, and the GS.sim scores were estimated.

The GS.CoMoD method is based on the assumption that if two semantic profiling vectors of disease genes that are obtained by gene-set enrichment analysis using functional gene sets show substantial similarity, the diseases are likely to be comorbid. The results of the simulation analysis validated this assumption. As described in the Methods, disease genes involved in type 2 diabetes were selected from the DisGeNET database because of their large number (*n* = 3134). The splitting of disease genes and the estimation of scores were repeated 1000 times (200 times per splitting ratio). The assignment of genes into two groups with an equal number of genes yielded a GS.sim score with higher similarity, whereas random disease genes exhibited lower GS.sim scores (Figure 2). The GS.sim scores were significantly different between the simulation settings (paired *t*-test, *p* < 1 × 10^−16^). Because the genes associated with type 2 diabetes were divided into two groups, if the assumption of GS.CoMoD is valid, then the change in the *p* values of the gene-set enrichment analysis should be similar. The results indicated that the similarity was substantial, which suggests that the semantic profiling vectors are representative of the biological mechanisms of diseases. The results of the simulation based on the random partitioning of genes of single diseases with different proportions supported the assumption of GS.CoMoD. The unequal distribution of the genes involved in a single disease into two groups and the subsequent application of GS.CoMoD revealed that the GS.sim scores were significantly higher than those of random disease genes, and the results were consistent regardless of the partitioning ratios (paired *t*-test, *p* < 1 × 10^−16^, Figure 2). These results were consistent when the simulation was performed using GOMF and Reactome gene sets (Figure S1). To identify potential bias when the number of disease genes was small, 20 genes associated with gingival fibromatosis–progressive deafness syndrome were also used in the simulation. The results showed the same tendency: even though the number of genes was small, the GS.sim scores from splitting the disease genes were greater than those from splitting random genes (Figure S2).

The simulation was repeated using randomly selected gene sets from the DisGeNET database [10]. Among the 30,293 disease gene sets of the DisGeNET database, 100 gene sets encompassing more than 10 genes were randomly selected. The results of this analysis were similar to those of the simulation using type 2 diabetes genes (Figure 2). The mean GS.sim scores were greater than those obtained from a simulation using the randomly selected disease genes, which was statistically significant (paired *t*-test, *p* value < 1 × 10^−16^). These results were consistent with the different functional gene sets (Figure S3). These findings suggest that the GS.sim score is valid for the determination of comorbidity using disease gene information.

### 2.3. Performance of GS.CoMoD in the Prediction of Comorbidity

#### 2.3.1. Comparison with Disease Comorbidity Measured Using the PPI Network

In Menche et al.’s study (MS), the authors developed the Sab measure, which can determine the comorbidity of diseases based on the distances of protein pairs in the PPI network [7]. The method computes distances within a gene set and between different gene sets of diseases. The differences between the shortest distances between the genes of two gene sets and the shortest distances between the genes within each gene set were used to measure the degree of separation between disease gene sets. The Sab measure was originally designed based on the distances of protein pairs, and even with no overlapping genes, it can detect comorbidity. The Supplementary Materials provide the disease gene data used in their analysis, as well as the Sab values of all pairs between disease gene sets; these data were used directly in the comparative analysis. Using these data, a comparative analysis was performed with GS.CoMoD, Sab, and two overlapping-based measures, i.e., the Jaccard index and the overlapping coefficient (Materials and Methods).

MS used 299 disease gene sets that were collected from the GWAS catalog [11] and OMIM databases [12]. The disease names were coded using MeSH terms, and the member genes were annotated by NCBI gene numbers; therefore, the corresponding gene symbols were matched using a mapping table that was downloaded from the HGNC database [13]. For the comparison of different comorbidity measures, it is necessary to determine the gold standards of comorbidity between diseases. The information was obtained from the diagnosis information of the HIRA 2009 data. The comorbidities were confirmed via the statistical testing of RRs that were computed using the riskratio function of the epitools R package [14]. Because the diagnoses included in the HIRA data were coded according to ICD 10 terms, it was necessary to map ICD 10 codes to MeSH disease terms. Using the DO, 1777 mappings between MeSHs and the ICD 10 were obtained. Among the 299 MeSH terms used in MS, 88 terms were mapped to ICD 10 terms (Table S1). Consequently, the comorbidity of 3828 disease pairs was tested using the HIRA 2009 and disease gene data.

Overlapping-based measures were included in the comorbidity analysis to compare the performance of the detection of comorbidity with or without the use of common genes between diseases. The Jaccard index and overlap coefficient were applied to measure the similarity using disease gene information. To determine the performance of the benchmark measures in the determination of comorbidity, the area under the curve (AUC) was estimated using the pROC R package [15]. When disease comorbidity was determined based on the RRs from the HIRA 2009 data, the *p*-value threshold was adjusted using Bonferroni’s multiple correction (=0.05/3828 = 1.31 × 10^−5^). Significant comorbidity was confirmed using two criteria: (1) a significant RR with the adjusted *p* value and (2) an RR > 1. In addition, an RR > 5 and RR > 10 were also used for the determination of comorbidity, and three different class labels were used in the comparison of the performance (Table S2).

The comparison of the performance of the measures revealed that the AUC of all measures generally increased as the thresholds of the RR increased. Among the measures tested, the GS.sim scores that were estimated with the GOBP showed the best performance at all RR thresholds (Table 1). Using the comorbidity class label of an RR > 10 threshold, the GS.sim score with the GOBP exhibited the best performance (AUC = 0.77). Although the GS.sim scores estimated with GOMF and Reactome gene sets showed comparable performance to those of the other measures, only the GS.sim scores estimated with the GOBP outperformed all measures consistently. For the comparison of the performance, disease pairs that had no common disease genes were selected, and the prediction performance was compared independently (Table 1). As expected, the overlapping-based measures (Jaccard index and overlap coefficient) exhibited poor performance (AUC = 0.50). The GS.sim score also had the best performance, similar to that observed in the prediction of total disease comorbidities. However, the GS.sim scores with different functional gene sets showed the best performance according to the thresholds of the RRs. For example, when the RR > 10, GS.sim scores using Reactome gene sets had the best performance, whereas the performance was greatest when the GS.sim scores were estimated using GOMF in cases with a threshold of RR > 1. In both conditions, the GS.sim scores had the best performance among all measures tested here.

#### 2.3.2. Comparison with Disease Comorbidity Determined Using Interactome and Functional Gene Sets

MS used only the PPI network for the determination of disease comorbidity, whereas Rubio-Perez et al.’s study (RPS) integrated various resources including an interactome, a network-based disease prediction method, and functional gene set data [8]. These authors adopted the strategy of expanding disease genes by integrating known and predicted disease genes and performed a gene-set enrichment analysis. The significant results under predefined thresholds were exclusively summed to yield final scores, which was different from the current approach in that no filtering of *p* values was performed. As in MS, these authors provided 234 MeSH terms with corresponding disease genes, which were used in their analysis. In that study, disease genes from the DisGeNET database were used, and two different versions of the DisGeNET database were applied in the analysis [8]. For comparison analysis, the composite scores developed by RPS were used directly, and disease genes from version 1 of the DisGeNET database were used in the analysis of GS.CoMoD and overlapping-based measures [16]. As in the comparative analysis with the work in MS, the MeSH terms were mapped to the ICD 10 codes: 85 MeSH terms were mapped to the ICD codes (Table S3), and 3570 disease pairs were included in the analysis. When no composite score was assigned, the scores were set to zero. The determination of the gold standards of disease comorbidity was performed using the same criteria (*p* value and RR thresholds of 1, 5, and 10; Table S4).

The results of the comparative analysis indicated that GS.CoMoD outperformed the other measures. For all RR thresholds with or without common genes, the GS.sim score outperformed the other measurers, with the Jaccard index showing the best performance only in cases with an RR > 5 (Table 2); otherwise, the GS.sim score with the GOBP outperformed all other measures. In turn, the strict and relaxed genetic measures reported by Rubio-Perez et al. exhibited poor performance. Considering that the relaxation of *p* values (relaxed measure) improved the performance, the poor performance mentioned above may stem from the *p*-value-based selection and uncertainty of disease gene prediction using a network-based tool.

The comorbidity analysis was repeated using disease genes of the current version of the DisGeNET database to identify the effect of disease genes on the determination of comorbidity. There were large differences in gene numbers between the previous and current versions of the 85 disease gene sets (Table S5). When the current disease genes were used, the overall performance of the measures, including the overlap-based ones, increased significantly (Table S6). For example, the performance of the GS.sim score with GOMF exhibited an abrupt change in the mean AUC, from 0.72 to 0.77. Especially when detecting comorbidities with no common genes, the greatest AUC for an RR > 1 increased from 0.66 to 0.88. These results indicate that the composition of disease genes is a critical factor for enhancing the performance of the gene-based determination of disease comorbidity, regardless of the measuring method used.

#### 2.3.3. Logistic Regression Analysis with Combination of GS.sim Scores

In the comparative analysis, the GS.sim scores showed outperformance across a wide range of RR thresholds. To identify whether the combination of multiple GS.sim scores improves prediction performance, logistic regression analysis with the glm R function was applied. For each threshold of the RR, four different models had different combinations of GS.sim scores estimated by the functional gene sets (Table S7). With default parameters, models having the highest AUC were selected.

The logistic regression models outperformed all the single scores, including the GS.sim. Various combinations of the GS.sim scores were found to show the best performance for different RR thresholds. In MS, the model showed the best performance with all thresholds of the RR, and this result was consistent in disease pairs without overlapping genes (Table 1). The results of RPS showed the same tendency. The logistic regression model outperformed the single scores with all RR thresholds (Table 2). Moreover, the tendency was the same with the current version of disease genes (Table S6).

### 2.4. Identification of Core Gene Sets

A case study for identifying core gene sets applied in the GS.CoMoD analysis was performed using disease genes involved in type 2 diabetes and hypertension. It is well known that these two diseases have a strong tendency to occur simultaneously [17]. From the DisGeNET database, genes that are associated with type 2 diabetes (*n* = 3134) and hypertension (*n* = 445) were collected, with 309 overlapping genes between them. The GOBP was used for the functional gene sets of GS.CoMoD.

With all GOBP terms, the GS.sim score of type 2 diabetes and hypertension was 0.41. Of the 7658 GOBPs, the core subset (*n* = 1207) showed the greatest similarity (=1), whereas the remaining 6451 GOBPs exhibited a lower score (=0.30), which was also lower than the GS.sim score with total GOBPs. It should be noted that some of the 1207 GOBPs from the different semantic profiling vectors had significant *p* values with multiple testing correction (adjusted *p* value = 0.05/7658 = 6.53 × 10^−6^), whereas the remaining GOBPs of the core subset had no significant *p* values (Table 3 and Table S8). This implies that, although many of the *p* values of the core subset were statistically significant, the changing patterns of the *p*-value vectors were very similar within the core subset, which is consistent with the concept of GS.CoMoD.

The core subset included biologically plausible GOBPs that are consistent with the current knowledge of the pathophysiology of type 2 diabetes and hypertension. For example, the ‘programmed cell death’ GOBP was highly significant in both diseases (Table 3), and it is well known that pancreatic and endothelial cell death occur in type 2 diabetes and hypertension, respectively [18,19]. Therefore, this GOBP might be one of the main pathophysiologies of comorbidity in type 2 diabetes and hypertension. Finally, such processes may provide valuable information for the development of a more specific treatment strategy for patients with both diseases.

## 3. Discussion

The simulation and real data analysis results showed that GS.CoMoD had substantial power in detecting disease comorbidity. In the real data analysis, GS.CoMoD detected comorbid relationships between disease pairs, even when there were no overlapping genes. For example, in the results of MS, idiopathic orofacial dystonia (G244) and Parkinson’s disease (G20) showed no common genes between their respective gene sets. However, in the HIRA data, they exhibited comorbidity at various thresholds of relative risk (RR = 1, 5, and 10). While the overlapping measures such as JI and OC indicated zero similarity, and the Sab score was 0.16 (lower than the average Sab score), the GS.sim with GOBP scored 0.39, which was higher than the mean score of 0.31. It is well known that orofacial dystonia can occur in Parkinson’s disease [20]. This may be a side effect of levodopa treatment [21] or an unusual manifestation of Parkinson’s disease itself [22]. In either case, it is difficult to infer a genetic relationship using overlapping measures alone. However, GS.CoMoD yielded a higher GS.sim score, which was further validated through the HIRA data analysis. Another disease pair, type 2 diabetes (E11) and Parkinson’s disease (G20), also demonstrated the superior performance of GS.CoMoD in the absence of overlapping genes. Although the comorbidity between type 2 diabetes and Parkinson’s disease is known, the RR for this disease pair was relatively low. As a result, it was only considered a positive comorbidity case when the RR threshold was set to 1, suggesting a relatively weak comorbid relationship. Even in this case, GS.sim with GOBP produced a higher score (=0.31) than the average GS.sim score, while the overlapping-based measures were zero, and the Sab score was 0.16, lower than the average. Previous studies have suggested a biological basis for the comorbidity between type 2 diabetes and Parkinson’s disease. Insulin signaling is a well-known factor in the development of Parkinson’s disease in patients with type 2 diabetes. The neurodegeneration associated with Parkinson’s disease may result from insulin dysregulation, the worsening of amyloid pathology, and altered synaptic plasticity [23]. Additionally, abnormal insulin signaling, mitochondrial dysfunction, and neuroinflammation are common pathophysiological mechanisms shared by both diseases [24]. These findings support the comorbidity between Parkinson’s disease and type 2 diabetes, even in the absence of shared disease genes. In the results, there were also disease pairs that showed no evidence of comorbidity in the HIRA data but exhibited high GS.sim scores. For instance, asthma (J45) and ankylosing spondylitis (M45) showed a GS.sim score of 0.40, while the other measures showed low scores (JI, OC = 0; Sab = 0.04). Although the RR did not yield a significant result, a previous study reported a comorbid relationship between the two diseases [25]. This may suggest a weak comorbidity relationship, and further biological and epidemiological studies are needed to confirm this finding.

In determining comorbidity using disease genes, there was a potential bias related to the number of disease genes, as a greater number of genes could increase the likelihood of gene overlap. To assess this bias, correlations between all comorbidity scores and the number of disease genes were examined. The results indicated a tendency for comorbidity scores to correlate with the number of genes in disease pairs. Specifically, when the scores were correlated with the number of union genes in each disease pair, significant correlations were observed—especially for the overlapping-based methods such as the Jaccard index (JI) and overlapping coefficient (OC) (Tables S8 and S9). In the benchmark dataset, both measures showed higher correlations. When the number of disease genes exceeded the mean value, these correlations became more pronounced. In the dataset in MS, many disease pairs had no overlapping genes (*n* = 2066). Consequently, the correlation between the number of genes and the JI score was relatively low (=0.02). However, when disease pairs with a larger number of genes were considered, the JI score increased substantially (=0.1, Table S8). In contrast, GS.sim with GOBP showed negative correlations. In MS, the OC and Sab scores exhibited higher correlations (0.29 and 0.11, respectively) than the other measures, whereas the GS.sim scores using different gene sets showed weaker correlations (−0.03, 0.04, and 0.1 with GOBP, GOMF, and Reactome gene sets, Table S8). This trend was even more evident in the dataset in RPS, where most disease pairs had no overlapping genes. In RPS, overlapping-based measures showed stronger correlations than GS.sim measures across all comparisons. When the number of disease genes exceeded the mean value, the JI and OC scores decreased substantially, although they still remained higher than the GS.sim scores (Table S9). This might be related to the increase in the number of union genes, which could decrease the magnitude of overlap-based measures. While strict and relaxed measures showed the least correlation, their predictive performance was also poor (Table 2). These findings suggest that GS.sim measures are less affected by the number of disease genes compared to overlapping-based methods.

One of the limitations of this study is that disease comorbidity was determined solely using the 2009 HIRA data. It is possible that comorbidity patterns vary across different ethnicities; therefore, incorporating global comorbidity data across diverse populations could provide a more robust gold standard for disease comorbidity analysis. Another limitation lies in the use of epidemiological data to determine comorbidity, as it is difficult to identify the exact causes of disease co-occurrence. For instance, one disease may lead to the onset of another, yet both may be classified as comorbid based on cross-sectional epidemiologic data. Additionally, medications for one disease may induce the development of another, which is also hard to detect in snapshot epidemiological datasets. Lastly, various types of genomic data were not utilized in this study. With the increasing use of multi-omics data in disease research, such data are invaluable for uncovering disease mechanisms [26]. Although not used in this study, integrating multi-omics data in comorbidity analysis could enhance translational research, particularly in identifying the core mechanisms underlying disease pair development. These limitations should be considered in future research.

## 4. Materials and Methods

### 4.1. GS.CoMoD and Other Disease Comorbidity Analyses

For the gene-set enrichment analysis of GS.CoMoD, two categories of GO, including BP and MF terms, were used. The cellular component terms were not used because they provide cellular structural information rather than functional implications. The Reactome pathway gene sets were also used in this analysis. All data were downloaded from the MSigDB website [27].

To test the performance of GS.CoMoD, disease gene data from previous studies were used. Menche et al. reported disease modules that were segregated based on the topology of the PPI network [7]. These authors developed a PPI-based measurement of distances between diseases, which can be applied to the detection of comorbidity. They defined the network-based separation of a disease pair (Sab), *A* and *B*, using

(2) $$SAB\;\equiv \;\langle dAB\rangle \;-\;\frac{\langle dAA\rangle \;+\;\langle dBB\rangle }{2}$$

*SAB* compares the shortest distances between two proteins of a disease pair. *dAB* is the shortest distance between the proteins of diseases *A* and *B*. *dAA* and *dBB* indicate the shortest distances within each disease. In turn, Rubio-Perez et al. reported a comorbidity measure that was determined by integrating the interactome with GO [8]. In both studies, disease gene data were provided and applied to the comparative analysis.

For benchmark analysis, two different comorbidity measures that are based on overlapping genes were used. The number of common genes between different disease gene lists has been frequently used to identify comorbidity using disease gene information. It is easy to compute, and the interpretation of results is straightforward. The Jaccard index is the most frequently used measure. When there are two sets of genes (*A* and *B*), the Jaccard index is defined as follows:

(3) $$Jaccard\;Index\;=\;\frac{\left|A\cap B\right|}{\left|A\cup B\right|}$$

As seen in Equation (3), the Jaccard index becomes greater as the number of overlapping genes increases. The overlap coefficient (*OC*) uses the intersection of disease genes as the numerator like the Jaccard index; however, the denominator is the minimum of the numbers of the two disease gene sets (Equation (4).

(4) $$OC\;=\;\frac{\left|A\cap B\right|}{\min\left(\left|A\right|,\left|B\right|\right)}$$

### 4.2. Identification of Core Gene Sets in Disease Comorbidity

In the case of disease gene pairs that exhibit a substantial GS.sim score, it is possible that the *p* values of some gene sets show higher similarity, thus yielding a higher GS.sim score compared with the remaining gene sets. These gene sets can be interpreted as the core gene sets that are involved in the development of the comorbidity of the diseases. For the identification of such gene sets, a mixture model regression (MMR) analysis was applied. MMR identifies clusters of samples that have differentially fitted regression lines [28]. Here, the model used an expectation–maximization algorithm, and the estimation of the model parameters was iterated until no substantial changes in model fitness were observed (Supplementary Methods). The results of MMR are equivalent to those of clustering. After the application of the model, the overall group of samples was divided into clusters with different fitted regression lines. The MMR model was applied to the pair of *p*-value vectors, and the number of clusters was set to 2. Consequently, the total *p* values were divided into two groups with different GS.sim scores. The gene ontology biological processes (GOBPs) with *p*-value vectors indicating a higher GS.sim score were selected as the core gene sets. For the estimation of the MMR parameters, the FlexMix R package (ver. 2) was applied [29].

### 4.3. Simulation Study

To test whether the GS.sim scores represent disease comorbidity, a simulation study was performed based on the random sampling of known disease genes. First, a set of disease genes with a large number of genes was selected, and the disease genes were divided into two groups according to prefixed ratios. The two gene sets were considered as different sets of disease genes, and the GS.sim score between the simulated disease gene lists was determined. Because the simulated disease gene sets originated from the single disease gene list, the GS.sim scores were expected to be substantial, as the gene lists had a high probability of sharing the same pathophysiological mechanism, thus resulting in an increase in the similarities of the semantic profiling vectors from the two simulated disease gene sets. In addition, the same procedure was repeated using the human genes that were randomly sampled. Of the total human genes (*n* = 43,136 from HGNC database [13]), the same number of genes as that of the selected disease gene set were randomly selected and divided into two groups. In this simulation setting of null cases, the GS.sim scores were expected to be low, as there would be no shared pathophysiologic mechanisms between the simulated disease gene lists because of the random sampling.

In addition to the simulation, a different procedure was applied to selected disease gene sets for comorbidity analysis, as follows. The genes of each disease gene set were divided equally and treated as two different disease gene sets; the GS.sim scores between the simulated disease gene sets were determined. The simulations described above were performed using three different gene sets, including GOBPs, molecular function (GOMF), and the Reactome database [30,31]. In addition, the same number of genes in each gene set was sampled from human genes, and the GS.sim score was determined by splitting the random genes into two groups with an equal number of genes. The divided gene sets were considered null comorbid disease pairs.

### 4.4. Collection of Disease Genes and Comorbidity Data

For the simulation analysis, the disease genes were collected from the DisGeNET database, which provides various gene sets of diseases and phenotypes [10]. The database file containing disease gene information was downloaded from the DisGeNET website (www.disgenet.org).

The comorbidity of a disease pair is determined by testing whether the occurrence of the two diseases is independent. For this purpose, hospital or large-scale epidemiologic data including disease incidence information are frequently used. In this research, the gold standards of disease comorbidities were confirmed using Health Insurance and Assessment Service (HIRA) 2009 data [32]. These data include more than one million South Korean individuals with diagnosis information, sampled in 2009 (*n* = 1,116,040). For each sample, the data include a diagnosis vector for the status of 12,745 diseases (0: normal; 1: disease). To determine the comorbidity of a disease pair, the RR was estimated.

The identifiers of disease gene lists in the data for comparative analysis use medical subject heading (MeSH) terminologies [33]. Because the HIRA 2009 data use the international classification of diseases 10 (ICD 10) coding system (https://www.who.int/standards/classifications/classification-of-diseases, accessed on 1 March 2024), the MeSH terms should be mapped to the ICD 10 to determine whether diseases coded using MeSHs are comorbid. The mapping of MeSH terms to the ICD 10 was performed using the human disease ontology (DO) database [34]. If the DO terms exhibited cross-referencing of a MeSH and an ICD 10 term simultaneously, the two terms were mapped. Of the mapping results, the one-to-one cases were included for analysis.

## 5. Conclusions

In this research, a novel method of detecting comorbidity based on the gene-set-based semantic profiling of disease genes was proposed. Simulation and comparison analyses revealed that GS.CoMoD outperformed the other measures regardless of the existence of overlapping genes between the disease gene sets. The results were consistent with the different thresholds of RRs that were used in the determination of the gold standards of disease comorbidity. Given this, GS.CoMoD is expected to give invaluable information for further research into disease comorbidity.

## Figures and Tables

**Table 1:** Results of the comparative analysis with the study by Menche et al.

	RR Thres	JI	OC	Sab	GOBP	GOMF	Reactome	LR
Total (*n* = 3828)	1	0.69	0.67	0.70	0.72	0.72	0.70	0.73
	5	0.71	0.70	0.74	0.75	0.72	0.72	0.75
	10	0.74	0.73	0.75	0.77	0.72	0.75	0.78
No overlap (*n* = 2066)	1	0.50	0.50	0.51	0.58	0.60	0.54	0.63
	5	0.50	0.50	0.48	0.62	0.58	0.59	0.66
	10	0.50	0.50	0.60	0.68	0.49	0.70	0.73

Total, total disease pairs; *n*, number of disease pairs; No overlap, disease pairs having no common disease genes; RR Thres, relative risk threshold; Jaccard, Jaccard index; OC, overlap coefficient; Sab, separation measure by Menche et al.; GOBP, gene-set-based similarity (GS.sim) score with gene ontology biological process; GOMF, GS.sim score with gene ontology molecular function; Reactome, GS.sim score with Reactome database; LR, logistic regression.

**Table 2:** Results of the comparative analysis with the study by Rubio-Perez et al.

	RR Thres	JI	OC	Strict	Relax	GOBP	GOMF	Reactome	LR
Total (*n* = 3570)	1	0.65	0.65	0.50	0.54	0.70	0.66	0.69	0.71
	5	0.76	0.75	0.50	0.52	0.70	0.73	0.75	0.77
	10	0.76	0.75	0.50	0.53	0.80	0.77	0.78	0.82
No overlap (*n* = 351)	1	0.50	0.50	0.50	0.52	0.66	0.54	0.60	0.68
	5	0.50	0.50	0.50	0.47	0.61	0.55	0.59	0.64
	10	0.50	0.50	0.50	0.47	0.78	0.59	0.57	0.80

Strict: genetic measure of comorbidity with a strict *p*-value threshold; Relax: genetic measure of comorbidity with a relaxed *p*-value threshold. The remaining abbreviations are the same as those defined in Table 1.

**Table 3:** Core subset of gene ontologies determined via mixture model regression.

GOBP	T2DM_Pval	HTN_Pval
Homeostatic process	0	1.16 × 10^−120^
Response to oxygen containing compound	0	1.33 × 10^−111^
Response to endogenous stimulus	7.92 × 10^−294^	3.51 × 10^−86^
Cellular response to oxygen containing compound	5.40 × 10^−271^	9.64 × 10^−77^
Regulation of transport	3.02 × 10^−270^	1.84 × 10^−88^
Positive regulation of signaling	1.30 × 10^−269^	1.04 × 10^−68^
Regulation of cell population proliferation	4.10 × 10^−268^	8.93 × 10^−70^
Programmed cell death	1.40 × 10^−246^	1.45 × 10^−50^
Regulation of cell death	1.10 × 10^−242^	2.44 × 10^−53^
Chemical homeostasis	4.02 × 10^−242^	4.17 × 10^−106^

GOBP, gene ontology biological process; T2DM, type 2 diabetes mellitus; HTN, hypertension; Pval, *p* value. Total GOs are listed in Table S8.

## Data Availability

Data is contained within the article and supplementary material.

## Supplementary Materials

The supporting information can be downloaded at https://www.mdpi.com/article/10.3390/ijms26083906/s1. References [9,10,29,35] are cited in the supplementary materials.
