# Supplementary material for: Measurement of Disease Comorbidity Using Semantic Profiling of Disease Genes
Source: Int J Mol Sci. 2025 Apr 21;26(8):3906. doi: 10.3390/ijms26083906 (PMC12028026; doi:10.3390/ijms26083906)
Supplement: Supplementary file 1 [file ijms-26-03906-s001.zip › Supplementary Methods & Results.docx]

**Supplementary Methods**

**Overrepresentation analysis**

Gene set analysis can be categorized into two forms: first, a model testing the enrichment for genes having the same biological implications, and second, the application and modification of a multivariate statistical model to sum up profiles of high-throughput experiments of multiple genes of a gene set. Overrepresentation analysis (ORA) belongs to the first gene set analysis category [1]. For this analysis, Fisher’s exact test was applied. After obtaining a list of disease genes, genes of gene ontologies (GOs) or pathways were identified in the disease genes. ORA tests whether more genes of a GO or pathway are included in the list than by random chance.

|  | Disease gene (+) | Disease gene (–) |  |
| --- | --- | --- | --- |
| GO genes (+) | *a* | *b* | *a*+*b* |
| GO genes (–) | *c* | *d* | *c*+*d* |
|  | *a*+*c* | *b*+*d* | *a*+*b*+*c*+*d* (= *n*) |

In the above table, the positive sign indicates that a gene is included in the list; the negative sign means the gene is not. *n* indicates the total number of human genes. Fisher’s exact test identifies whether the distribution of numbers in the table results from random chance. TheP-value of the test is obtained using the hypergeometric distribution, as shown in equation (1).

(1)

In the ORA, a one-sided test was performed to evaluate the alternative hypothesis that more genes of a functional gene set (GO or pathway) are included in the list than by random chance. The fisher.test function of the R program was used for this purpose, and the alternative parameter was set to greater for the one-sided testing.

**Collection of disease genes**

Disease genes were downloaded from the DisGeNET website [2]. The disease gene sets were stored in the form of an SQLite database file, and each database table was extracted using the RSQLite library [3]. The gene symbol was used for disease gene annotation, and the name of the diseases was coded with the Unified Medical Language System (UMLS) nomenclature. In total, 30,293 gene sets were associated with diseases or phenotypes.

**Mixture model regression for identification of a core subset**

Mixture model regression (MMR) is a statistical model that uses the expectation-maximization (EM) algorithm to estimate the multiple regression models that have the best fit for data [4]. Usually, a single regression model is estimated for fitting total data. However, in the MMR model, data are clustered into several groups, and different regression models are estimated in each group. The estimation of model parameters and cluster memberships is performed using the EM algorithm. In the MMR, the model is defined as follows.

(2)

(3)

*Κ* indicates several clusters, and y is a dependent variable with a conditional density of *h*. *Χ* is a vector containing independent variables, and *π*k is a prior probability of cluster *Κ*. The mixture of linear regressions with single variables was estimated. In this condition, f is a univariate normal distribution with cluster-specific mean and variance . The mixture of linear regression can be represented as follows.

(4)

In equation (4), y*i* is the value of the response variable in the i*th* observation. In the estimation step of the EM algorithm, the posterior probability of an instance comes from class *j* and is given by

, (5)

where *ψ* is a vector of *π* and *θ* of all clusters. According to the posterior probabilities, instances are mapped to specific clusters. The log-likelihood of a sample of N observations is determined by equation (6).

(6)

The parameters maximizing the equation cannot be estimated directly, and the iterative EM algorithm is applied to estimate parameters. In the maximization step of the EM, posterior probabilities of each observation are used to derive prior class probabilities (equation 7).

(7)

(8)

In addition, parameters that maximize the log-likelihood for each cluster are estimated.

(9)

In the linear regression, the parameters are updated as follows.

(10)

(11)

(12)

In the application of MMR, the intercept term was deleted to approximate the correlation coefficients. The MMR estimates the regression line between the semantic profiling vectors. Because the correlation coefficients of the vectors are not the same as the estimates of the regression line, the MMR analysis did not include an intercept term to approximate the correlation coefficients.

**Reference**

1. Maleki,F. et al. (2020) Gene set analysis: challenges, opportunities, and future research. Front. Genet., 11, 654.

2. Pinero,J. et al. (2020) The DisGeNET knowledge platform for disease genomics: 2019 update. Nucleic Acids Res., 48(D1), D845–D855.

3. Müller K. et al. (2022) RSQLite: SQLite Interface for R. https://rsqlite.r-dbi.org, https://github.com/r-dbi/RSQLite.

4. Grun,B. and Leisch,F. (2008) FlexMix Version 2: finite mixtures with concomitant variables and varying and constant parameters. J. Stat. Softw., 28(4), 1–35.

**Supplementary Table 1**. Mapping between MeSH and ICD10 in Menche’s data

| Name | MeSH | ICD10 |
| --- | --- | --- |
| alzheimer disease | MESH:D000544 | G30 |
| amino acid metabolism, inborn errors | MESH:D000592 | E729 |
| anemia | MESH:D000740 | D649 |
| anemia, aplastic | MESH:D000741 | D619 |
| ankylosis | MESH:D000844 | M246 |
| arteriosclerosis | MESH:D050197 | I70 |
| arthritis | MESH:D001168 | M1990 |
| arthritis, rheumatoid | MESH:D001172 | M069 |
| asthma | MESH:D001249 | J45 |
| bacterial infections | MESH:D001424 | A499 |
| behcet syndrome | MESH:D001528 | M352 |
| biliary tract diseases | MESH:D001660 | K839 |
| blood coagulation disorders | MESH:D001778 | D689 |
| bone diseases | MESH:D001847 | M899 |
| breast diseases | MESH:D001941 | N649 |
| breast neoplasms | MESH:D001943 | C50 |
| cardiomyopathies | MESH:D009202 | I42 |
| cardiomyopathy, dilated | MESH:C580047 | I420 |
| celiac disease | MESH:D002446 | K900 |
| central nervous system diseases | MESH:D002493 | G969 |
| cerebrovascular disorders | MESH:D002561 | I679 |
| charcot-marie-tooth disease | MESH:C537985 | G600 |
| cholestasis | MESH:D002779 | K831 |
| colitis | MESH:D003092 | K529 |
| colitis, ulcerative | MESH:D003093 | K51 |
| colorectal neoplasms | MESH:D015179 | C189 |
| cranial nerve diseases | MESH:D003389 | G529 |
| crohn disease | MESH:D003424 | K501 |
| diabetes mellitus, type 1 | MESH:D003922 | E10 |
| diabetes mellitus, type 2 | MESH:D003924 | E11 |
| digestive system diseases | MESH:D004066 | K929 |
| dyskinesias | MESH:D020820 | G244 |
| endocrine gland neoplasms | MESH:D004701 | C759 |
| endocrine system diseases | MESH:D004700 | E349 |
| esophageal diseases | MESH:D004935 | K229 |
| eye diseases | MESH:D005128 | H44 |
| genital diseases, male | MESH:D005832 | N509 |
| genital neoplasms, male | MESH:D005834 | C639 |
| nephritis | MESH:D009393 | N08 |
| goiter | MESH:D006042 | E049 |
| graves disease | MESH:D006111 | E050 |
| heart arrest | MESH:D006323 | I46 |
| heart defects, congenital | MESH:D006330 | Q249 |
| heart diseases | MESH:D006331 | I519 |
| hematologic diseases | MESH:D006402 | D759 |
| hemorrhagic disorders | MESH:D006474 | D699 |
| hyperinsulinism | MESH:D006946 | E161 |
| hyperthyroidism | MESH:D013971 | E059 |
| hypothalamic diseases | MESH:D007027 | E236 |
| immunologic deficiency syndromes | MESH:D007153 | D849 |
| immunoproliferative disorders | MESH:D007160 | C889 |
| intestinal diseases | MESH:D007410 | K639 |
| intestinal neoplasms | MESH:D007414 | C260 |
| kidney neoplasms | MESH:D007680 | C64 |
| leukemia, lymphoid | MESH:D007945 | C91 |
| leukemia, myeloid | MESH:D007951 | C931 |
| leukemia, myeloid, acute | MESH:D015470 | C920 |
| lipidoses | MESH:D008064 | E756 |
| liver diseases | MESH:D008107 | K769 |
| lung diseases | MESH:D008171 | J984 |
| lupus erythematosus, systemic | MESH:D008180 | M329 |
| lymphoma | MESH:D008223 | C859 |
| metabolic diseases | MESH:D008659 | E889 |
| multiple sclerosis | MESH:D009103 | G35 |
| muscular diseases | MESH:D009135 | G729 |
| muscular dystrophies | MESH:D009136 | G710 |
| myeloproliferative disorders | MESH:D009196 | D471 |
| myocardial infarction | MESH:D009203 | I21 |
| nervous system diseases | MESH:D009422 | G98 |
| neurodegenerative diseases | MESH:D019636 | G319 |
| neuromuscular diseases | MESH:D009468 | G709 |
| obesity | MESH:D009765 | E669 |
| orbital diseases | MESH:D009916 | H059 |
| osteochondrodysplasias | MESH:D010009 | Q789 |
| ovarian neoplasms | MESH:D010051 | C56 |
| parkinson disease | MESH:D010300 | G20 |
| prostatic diseases | MESH:D011469 | N429 |
| prostatic neoplasms | MESH:D011471 | C61 |
| proteinuria | MESH:D011507 | R80 |
| psoriasis | MESH:D011565 | L403 |
| retinal diseases | MESH:D012164 | H359 |
| spastic paraplegia, hereditary | MESH:C565409 | G114 |
| spinal cord diseases | MESH:D013118 | G959 |
| spondylitis | MESH:D013166 | M46 |
| spondylitis, ankylosing | MESH:D013167 | M45 |
| thoracic neoplasms | MESH:D013899 | C761 |
| thyroid diseases | MESH:D013959 | E079 |
| uveitis | MESH:D014605 | H209 |

MESH, medical subject headings; ICD10, international classification of diseases 10.

**Supplementary Table 2.** Number of instances in comorbidity and non-comorbidity disease pairs in Menche *et al*.’s data.

|  | RR threshold | Comorbidity (-) | Comorbidity (+) |
| --- | --- | --- | --- |
| Total  (*n* = 3828) | RR>1 | 3774 | 54 |
|  | RR>5 | 3794 | 34 |
|  | RR>10 | 3805 | 23 |
| Overlap (-)  (*n* = 2066) | RR>1 | 2050 | 16 |
|  | RR>5 | 2056 | 10 |
|  | RR>10 | 2061 | 5 |

Total, total disease pairs; Overlap (-), disease pairs having no common genes; n, number of disease pairs, RR; relative risk.

**Supplementary Table 3.** Results of identifier mapping in Rubio-Perez et al. data.

| MESH | UMLS | ICD10 |
| --- | --- | --- |
| MESH:D008288 | C0024530 | B54 |
| MESH:D003110 | C0007102 | C18 |
| MESH:D015179 | C0009402 | C189 |
| MESH:D012004 | C0007113 | C20 |
| MESH:D006528 | C2239176 | C220 |
| MESH:D007414 | C0021841 | C260 |
| MESH:D008654 | C0025500 | C45 |
| MESH:D001943 | C0006142 | C50 |
| MESH:D002583 | C0007873 | C53 |
| MESH:D016889 | C0014170 | C541 |
| MESH:D014594 | C0042138 | C55 |
| MESH:D010051 | C0919267 | C56 |
| MESH:D011471 | C0033578 | C61 |
| MESH:D001749 | C0005684 | C67 |
| MESH:D013964 | C0040136 | C73 |
| MESH:D008223 | C0024299 | C859 |
| MESH:D009101 | C0026764 | C900 |
| MESH:D015470 | C0023467 | C920 |
| MESH:D015473 | C0023487 | C924 |
| MESH:D000756 | C0002896 | D643 |
| MESH:D000740 | C0002871 | D649 |
| MESH:D007153 | C0003257 | D849 |
| MESH:D007037 | C0020676 | E039 |
| MESH:D006111 | C0018213 | E050 |
| MESH:D013967 | C0920350 | E063 |
| MESH:D003924 | C0011860 | E11 |
| MESH:D011085 | C0032460 | E282 |
| MESH:D016649 | C0085215 | E283 |
| MESH:D009765 | C1563743 | E669 |
| MESH:D000592 | C0002514 | E729 |
| MESH:D008064 | C0023794 | E756 |
| MESH:D019189 | C0012715 | E831 |
| MESH:D000686 | C0002726 | E85 |
| MESH:D000437 | C0001956 | F101 |
| MESH:D012559 | C0036341 | F20 |
| MESH:D001714 | C0005586 | F31 |
| MESH:D001008 | C0003469 | F419 |
| MESH:D009771 | C0028768 | F42 |
| MESH:D001321 | C0004352 | F840 |
| MESH:D010300 | C0030567 | G20 |
| MESH:D000544 | C0002395 | G30 |
| MESH:D019636 | C0270715 | G319 |
| MESH:D009103 | C0026769 | G35 |
| MESH:D008881 | C0007852 | G43 |
| MESH:D002607 | C0007959 | G600 |
| MESH:D009468 | C0002735 | G709 |
| MESH:D002386 | C0086543 | H26 |
| MESH:D006973 | C0020538 | I10 |
| MESH:D009203 | C0027051 | I21 |
| MESH:D009202 | C0033141 | I42 |
| MESH:D002311 | C0007193 | I420 |
| MESH:D006331 | C0018799 | I519 |
| MESH:D050197 | C0004153 | I70 |
| MESH:D029424 | C0024117 | J449 |
| MESH:D001249 | C0004096 | J45 |
| MESH:D008171 | C4021760 | J984 |
| MESH:D009914 | C0029172 | K135 |
| MESH:D013276 | C0038358 | K25 |
| MESH:D006548 | C0019284 | K44 |
| MESH:D003424 | C0010346 | K501 |
| MESH:D003093 | C0009324 | K51 |
| MESH:D008107 | C0023895 | K769 |
| MESH:D002779 | C0008370 | K831 |
| MESH:D002446 | C0007570 | K900 |
| MESH:D003876 | C0011615 | L20 |
| MESH:D011565 | C0030246 | L403 |
| MESH:D001172 | C0003873 | M069 |
| MESH:D008180 | C0024141 | M329 |
| MESH:D010024 | C0029459 | M810 |
| MESH:D007674 | C0022658 | N08 |
| MESH:D007248 | C0021364 | N46 |
| MESH:D011225 | C0032914 | O14 |
| MESH:D008831 | C0025958 | Q02 |
| MESH:D016142 | C0078982 | Q042 |
| MESH:D016135 | C0038219 | Q05 |
| MESH:D003103 | C0009363 | Q130 |
| MESH:D006330 | C0018798 | Q249 |
| MESH:D002972 | C0008925 | Q35 |
| MESH:D002971 | C0008924 | Q36 |
| MESH:D006627 | C0019569 | Q431 |
| MESH:D010009 | C0013366 | Q789 |
| MESH:D014983 | C0043346 | Q821 |
| MESH:D008382 | C0024796 | Q874 |
| MESH:D004314 | C0013080 | Q90 |
| MESH:D006943 | C0020456 | R739 |

MESH, medical subject headings, UMLS, unified medical language system, ICD10, international classification of diseases 10

**Supplementary Table 4.** Number of instances in comorbidity and non-comorbidity disease pairs in Rubio-Perez *et al*.’s data.

|  | RR threshold | Comorbidity (-) | Comorbidity (+) |
| --- | --- | --- | --- |
| Total  (*n* = 3570) | RR>1 | 3479 | 91 |
|  | RR>5 | 3537 | 33 |
|  | RR>10 | 3548 | 22 |
| Overlap(-)  (*n* = 2995) | RR>1 | 2945 | 50 |
|  | RR>5 | 2983 | 12 |
|  | RR>10 | 2987 | 8 |
| Overlap(-) with V3  (*n* = 351) | RR>1 | 347 | 4 |

The number of overlapping genes were determined with genes of DisGeNET version 1. When the numbers were determined with gene sets from current version (V3, downloaded on December, 2022) of DisGeNET database, no significant disease pairs were included with the thresholds of RR>5, and RR>10. This results from updating disease genes in the database, which leads to the significant decline in the number of disease pairs with no overlapping genes (*n* = 351). Total, total disease pairs; Overlap (-), disease pairs having no common genes; *n*, number of disease pairs; RR, relative risk.

**Supplementary Table 5.** Number of genes in disease gene sets from DisGeNET version 1 and current version.

|  | V1 | V3 |
| --- | --- | --- |
| MESH:D008288 | 21 | 685 |
| MESH:D003110 | 145 | 2969 |
| MESH:D015179 | 83 | 5472 |
| MESH:D012004 | 6 | 472 |
| MESH:D006528 | 40 | 5725 |
| MESH:D007414 | 5 | 182 |
| MESH:D008654 | 28 | 560 |
| MESH:D001943 | 272 | 6940 |
| MESH:D002583 | 5 | 140 |
| MESH:D016889 | 12 | 261 |
| MESH:D014594 | 4 | 32 |
| MESH:D010051 | 28 | 2542 |
| MESH:D011471 | 338 | 1722 |
| MESH:D001749 | 29 | 2113 |
| MESH:D013964 | 7 | 1164 |
| MESH:D008223 | 6 | 1548 |
| MESH:D009101 | 10 | 1740 |
| MESH:D015470 | 21 | 3111 |
| MESH:D015473 | 11 | 651 |
| MESH:D000756 | 4 | 22 |
| MESH:D000740 | 5 | 847 |
| MESH:D007153 | 7 | 21 |
| MESH:D007037 | 6 | 613 |
| MESH:D006111 | 5 | 585 |
| MESH:D013967 | 4 | 161 |
| MESH:D003924 | 52 | 3134 |
| MESH:D011085 | 4 | 988 |
| MESH:D016649 | 6 | 333 |
| MESH:D009765 | 48 | 4 |
| MESH:D000592 | 8 | 20 |
| MESH:D008064 | 12 | 44 |
| MESH:D019189 | 4 | 15 |
| MESH:D000686 | 4 | 694 |
| MESH:D000437 | 7 | 218 |
| MESH:D012559 | 76 | 2879 |
| MESH:D001714 | 6 | 1201 |
| MESH:D001008 | 7 | 840 |
| MESH:D009771 | 4 | 175 |
| MESH:D001321 | 25 | 1112 |
| MESH:D010300 | 28 | 2078 |
| MESH:D000544 | 29 | 3397 |
| MESH:D019636 | 6 | 43 |
| MESH:D009103 | 12 | 1800 |
| MESH:D008881 | 5 | 9 |
| MESH:D002607 | 6 | 208 |
| MESH:D009468 | 4 | 2 |
| MESH:D002386 | 10 | 878 |
| MESH:D006973 | 41 | 2322 |
| MESH:D009203 | 24 | 1800 |
| MESH:D009202 | 7 | 108 |
| MESH:D002311 | 22 | 512 |
| MESH:D006331 | 13 | 537 |
| MESH:D050197 | 7 | 2044 |
| MESH:D029424 | 5 | 1428 |
| MESH:D001249 | 47 | 2096 |
| MESH:D008171 | 8 | 1 |
| MESH:D009914 | 14 | 106 |
| MESH:D013276 | 4 | 136 |
| MESH:D006548 | 8 | 78 |
| MESH:D003424 | 30 | 1382 |
| MESH:D003093 | 19 | 1458 |
| MESH:D008107 | 14 | 1019 |
| MESH:D002779 | 7 | 420 |
| MESH:D002446 | 12 | 527 |
| MESH:D003876 | 5 | 751 |
| MESH:D011565 | 16 | 74 |
| MESH:D001172 | 41 | 2722 |
| MESH:D008180 | 24 | 1883 |
| MESH:D010024 | 15 | 73 |
| MESH:D007674 | 15 | 1180 |
| MESH:D007248 | 9 | 516 |
| MESH:D011225 | 7 | 166 |
| MESH:D008831 | 7 | 1064 |
| MESH:D016142 | 7 | 35 |
| MESH:D016135 | 6 | 12 |
| MESH:D003103 | 4 | 129 |
| MESH:D006330 | 5 | 406 |
| MESH:D002972 | 8 | 611 |
| MESH:D002971 | 5 | 255 |
| MESH:D006627 | 8 | 384 |
| MESH:D010009 | 11 | 24 |
| MESH:D014983 | 7 | 137 |
| MESH:D008382 | 4 | 130 |
| MESH:D004314 | 7 | 766 |
| MESH:D006943 | 4 | 1098 |

V1: DisGeNET version 1, V3: current version of DisGeNET.

**Supplementary Table 6**. Result of comparative analysis with Rubio-Perez data and current version of DisGeNET database.

|  | RR thres | JI | OC | Strict | Relax | GOBP | GOMF | Reactome | LR |
| --- | --- | --- | --- | --- | --- | --- | --- | --- | --- |
| Total (*n* = ) | RR_1 | 0.77 | 0.69 | 0.50 | 0.54 | 0.74 | 0.74 | 0.73 | **0.75** |
|  | RR_5 | 0.78 | 0.72 | 0.50 | 0.52 | 0.79 | 0.78 | 0.79 | **0.80** |
|  | RR_10 | 0.78 | 0.76 | 0.50 | 0.53 | 0.79 | 0.79 | 0.81 | **0.81** |
| No Overlap (*n* = ) | RR_1 | 0.50 | 0.50 | 0.50 | 0.52 | 0.86 | 0.88 | 0.76 | **0.90** |

Total, total disease pairs; n, number of disease pairs; No overlap, disease pairs having no common disease genes; RR thres, relative risk threshold; Jaccard, Jaccard index; OC, overlap coefficient; Strict, genetic measure of comorbidity with a strict *P* value threshold; Relax: genetic measure of comorbidity with a relaxed *P* value threshold; GS.CoM, gene-set-based comorbidity score; GOBP, gene ontology biological process; GOMF, gene ontology molecular function; Reactome, Reactome database

**Supplementary Table 7**. Model section of logistic regression analysis

| Data | Range of  disease pairs | RR  threshold | LR | Model |
| --- | --- | --- | --- | --- |
| Menche | Total | RR_1 | 0.7310 | GOBP + GOMF |
|  |  | RR_5 | 0.7528 | GOBP + GOMF |
|  |  | RR_10 | 0.7759 | GOBP + GOMF + Reactome |
|  | Overlap (-) | RR_1 | 0.6258 | GOBP + GOMF + Reactome |
|  |  | RR_5 | 0.6566 | GOBP + GOMF + Reactome |
|  |  | RR_10 | 0.7253 | GOBP + GOMF + Reactome |
| Rubio-Perez + DisGeNET V.1 | Total | RR_1 | 0.7129 | GOBP + Reactome |
|  |  | RR_5 | 0.7718 | GOMF + Reactome |
|  |  | RR_10 | 0.8169 | GOBP + GOMF + Reacome |
|  | Overlap (-) | RR_1 | 0.6771 | GOBP + GOMF |
|  |  | RR_5 | 0.6414 | GOBP + Reactome |
|  |  | RR_10 | 0.8025 | GOBP + Reactome |
| Rubio-Perez + DisGeNET V.3 | Total | RR_1 | 0.7459 | GOBP + GOMF + Reacome |
|  |  | RR_5 | 0.7963 | GOBP + Reacome |
|  |  | RR_10 | 0.8118 | GOBP + Reacome |
|  | Overlap (-) | RR_1 | 0.9035 | GOBP + GOMF |

GOBP, gene ontology biological process; GOMF, gene ontology molecular function; Reactome, Reactome database.

**Supplementary Table 8**. Correlation between measures and number of disease genes in Menche’s data

|  | JI | OC | Sab | GO.sim_GOBP | GO.sim_GOMF | GO.sim_Reactome |
| --- | --- | --- | --- | --- | --- | --- |
| Total pairs | 0.02 (0.13) | 0.29  (<2E-16) | 0.11 (1.08E-12) | -0.03 (0.07) | 0.04 (6.67E-03) | 0.1  (2.73E-09) |
| Pairs > (N = 198.1) | 0.11 (3.30E-06) | 0.29  (<2E-16) | 0.16 (1.57E-12) | -0.13 (5.15E-09) | -0.08 (1.00E-04) | 0.06  (1.00E-2) |

N: average number of union genes of disease pairs, JI: Jaccard Index, OC: overlap coefficient, GO.sim: gene set based similarity, GOBP: gene ontology biological process, GOMF: gene ontology molecular function. *P* values were presented within parenthesis.

**Supplementary Table 9**. Correlation between measures and number of disease genes in Rubio-Perez’s data

| N | JI | OC | Strict | Relax | GO.sim_GOBP | GO.sim_GOMF | GO.sim_Reactome |
| --- | --- | --- | --- | --- | --- | --- | --- |
| Total pairs | 0.24 (<2E-16) | 0.38  (<2E-16) | 0.03 (0.13) | 0.06 (1.01E-04) | 0.01  (0.40) | 0.06 (1.79E-04) | 0.09  (1.92E-07) |
| Pairs > (N = 45.8) | 0.07 (0.03) | 0.16 (3.32E-06) | -0.01 (0.63) | -0.04 (0.24) | 0.06  (0.05) | 0.08  (0.03) | 0.08  (0.02) |

N: average number of union genes of disease pairs, JI: Jaccard Index, OC: overlap coefficient, Strict: strict measure of comorbidity, Relax: relaxed measure of comorbidity, GO.sim: gene set based similarity, GOBP: gene ontology biological process, GOMF: gene ontology molecular function. *P* values were presented within parenthesis.

**Supplementary Fig. 1.** Results of simulation using known disease genes.


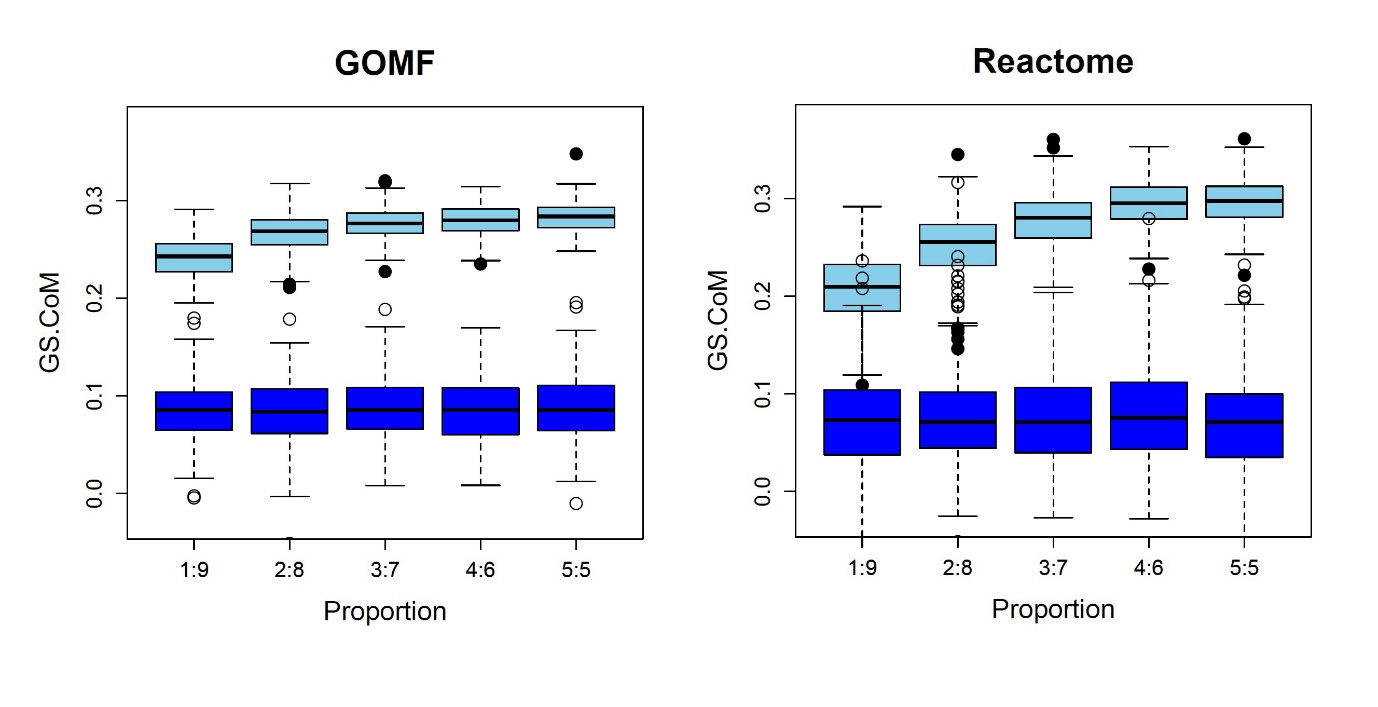


Note that GS.CoM score increased when the proportion changed to more equal splitting. GS.CoM, gene-set-based comorbidity

**Supplementary Fig. 2**. Results of simulation using known disease genes (C0016149: Gingival fibromatosis-progressive deafness syndrome).


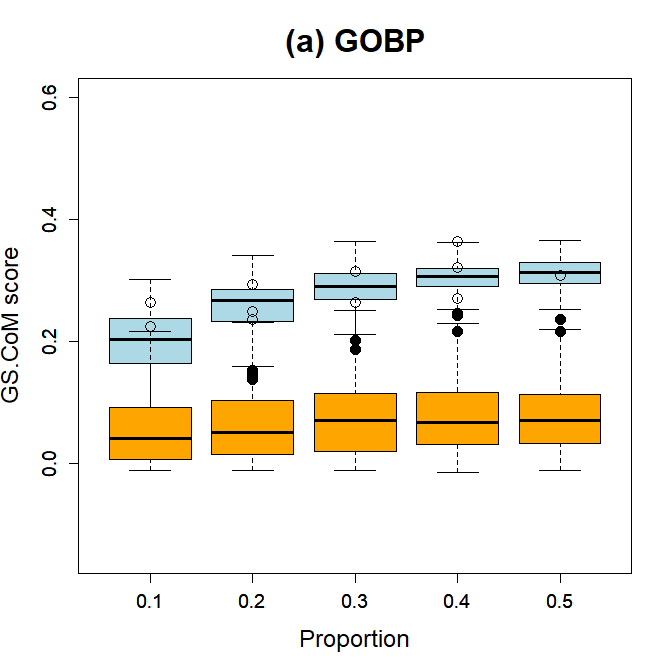

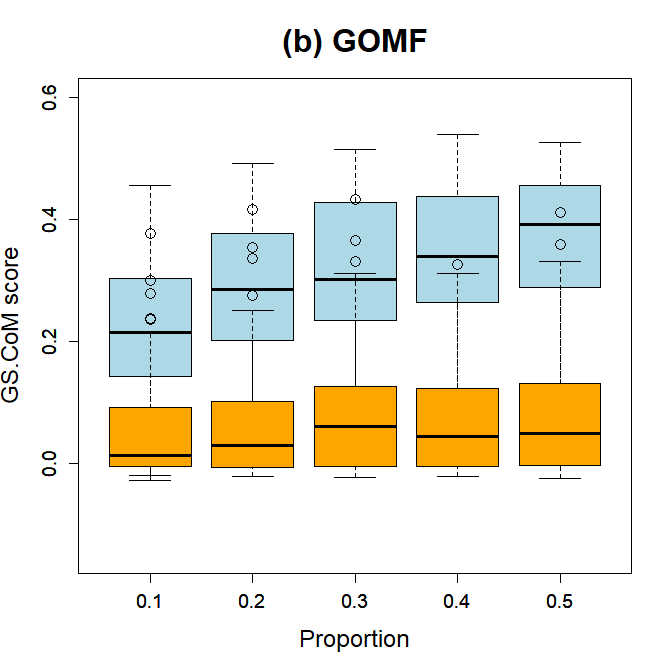

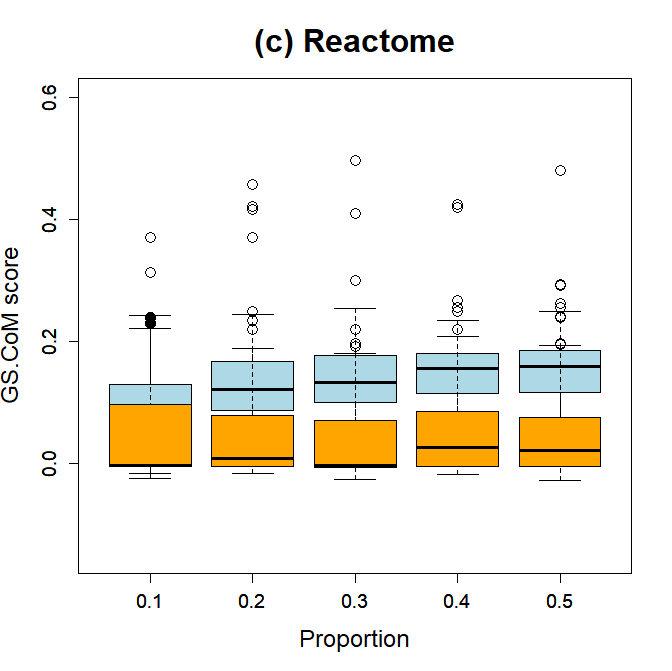


GS.CoM, gene set based comorbidity; GOMF, gene ontology molecular function; Reactome, Reactome database.


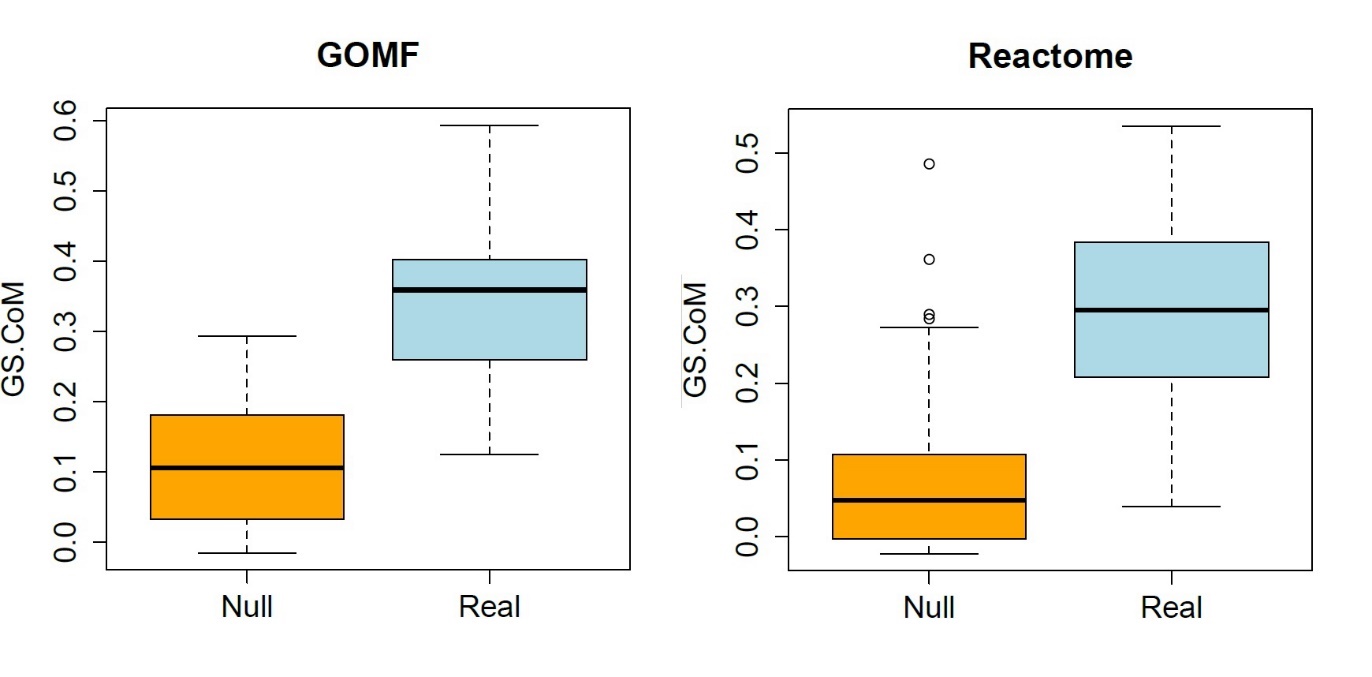
**Supplementary Fig. 3.** Results of simulation with randomly selected disease gene sets.

GS.CoM, gene set based comorbidity; GOMF, gene ontology molecular function; Reactome, Reactome database.
